# Human Respiratory Monitoring Based on Schottky Resistance Humidity Sensors

**DOI:** 10.3390/ma13020430

**Published:** 2020-01-16

**Authors:** Cunguang Lou, Kaixuan Hou, Weitong Zhu, Xin Wang, Xu Yang, Rihe Dong, Hongjia Chen, Linjuan Guo, Xiuling Liu

**Affiliations:** 1 Department of Biomedical Engineering, College of Electronic Information Engineering & Key Laboratory of Digital Medical Engineering of Hebei Province, Hebei University, Baoding 071002, Hebei, China; loucunguang@163.com (C.L.); HKxuan@yeah.net (K.H.); zhuweitong@live.com (W.Z.); wangxin_damon@163.com (X.W.); yangxuxu19970301@163.com (X.Y.); drh2232262371@163.com (R.D.); gaga1995716@163.com (H.C.); 2 College of Physical Science and Technology, Hebei University, Baoding 071000, Hebei, China

**Keywords:** Schottky structure sensor, resistance humidity sensor, human respiratory monitoring

## Abstract

Two types of Schottky structure sensors (silicon nanowire (SiNW)/ZnO/reduced graphene oxide (rGO) and SiNW/TiO_2_/rGO) were designed, their humidity resistance characteristics were studied, and the sensors were applied to detect sleep apnea through breath humidity monitoring. The results show that the resistance of the sensors exhibited significant changes with increasing humidity, the response times of the two sensors within the relative humidity range of 23–97% were 49 s and 67 s, and the recovery times were 24 s and 43 s, respectively. Meanwhile, continuous breathing monitoring results indicate that the sensitivity of the sensors remained basically unchanged during 10 min of normal breathing and simulated apnea. The response of the sensor is still good after 30 days of use. We believe that the Schottky structure composite sensor is a very promising technology for human breathing monitoring.

## 1. Introduction

Sleep apnea hypopnea syndrome (SAHS) is a prevalent and potentially serious disorder with the occurrence of at least 30 apneas during normal 7-hour sleep, where each apnea time is at least 10 s [1]. SAHS is characterized by recurrent apnea and sleep interruption during sleep, which result in a series of pathophysiological changes in the body. More and more patients suffer from SAHS complications, such as hypertension and stroke, and SAHS has become the second leading cause of stroke in particular [2]. Therefore, respiratory monitoring of individuals with SAHS diseases is particularly important and urgent. The moisture content in exhaled human breath is about 6%, and the use of humidity for breathing pattern monitoring may thus be an effective strategy.

With the rapid development of science and technology, more and more new materials have been developed for humidity-sensitive sensors in recent years, and composite nanomaterials have become a focus for research, including one-dimensional carbon, silicon, and semiconductor nanoparticles [3,4]. As a new type of semiconductor nanomaterial, silicon nanowire (SiNW) has the characteristics of large specific surface area, high surface activity, and high sensitivity to humidity, Yang designed a high-sensitivity SiNW array for humidity measurement [5]. Meanwhile, zinc oxide (ZnO) is an n-type semiconductor metal oxide with high electron mobility and stable chemical properties; it is very easy to adjust the surface [6,7], and it was demonstrated that ZnO is suitable for the detection of humidity. In addition, titanium dioxide (TiO_2_) nanomaterials can also be used as moisture-sensitive components [8] screen-printed TiO_2_ was proposed as the sensitive layer of a humidity sensor, and Milad Ghalamboran reported TiO_2_–SnO_2_ composites for moisture detection [9]. In addition, modified doped graphene (GO) sensors have fast response to humidity due to GO’s high charge carrier mobility and content of surface defects and residual oxygen-containing groups (carboxyl, epoxy, hydroxyl, etc.) [10,11,12,13]. Wearable electronic textile humidity sensors based on carbon nanotubes and composite fibers have also been developed [14,15]. The Schottky structure has been used extensively in photoelectric sensing, and Schottky structure humidity sensors for noncontact medical monitoring have been reported in recent years [16]. Inspired by the excellent performance of graphene and metal oxide, SiNW/ZnO/rGO and SiNW/TiO_2_/rGO Schottky composite metal nanomaterials were developed and used for sensitive detection of trace explosive vapors [17,18], and we found that their response to humidity is very sensitive and that they can be used for human respiration monitoring.

In this paper, we report two kinds of Schottky structure humidity sensors, SiNW/ZnO/rGO and SiNW/TiO_2_/rGO, which combine the advantages of graphene and metal oxide. We found that the resistance of the three-dimensional compound materials is sensitive to humidity, and the high sensitivity, fast response time, and excellent repeatability of the sensors provide high potential and very good prospects for the design of a portable human breathing monitoring system.

## 2. Experimental Details

### 2.1. Fabrication of the Sensor

**Figure 1:**
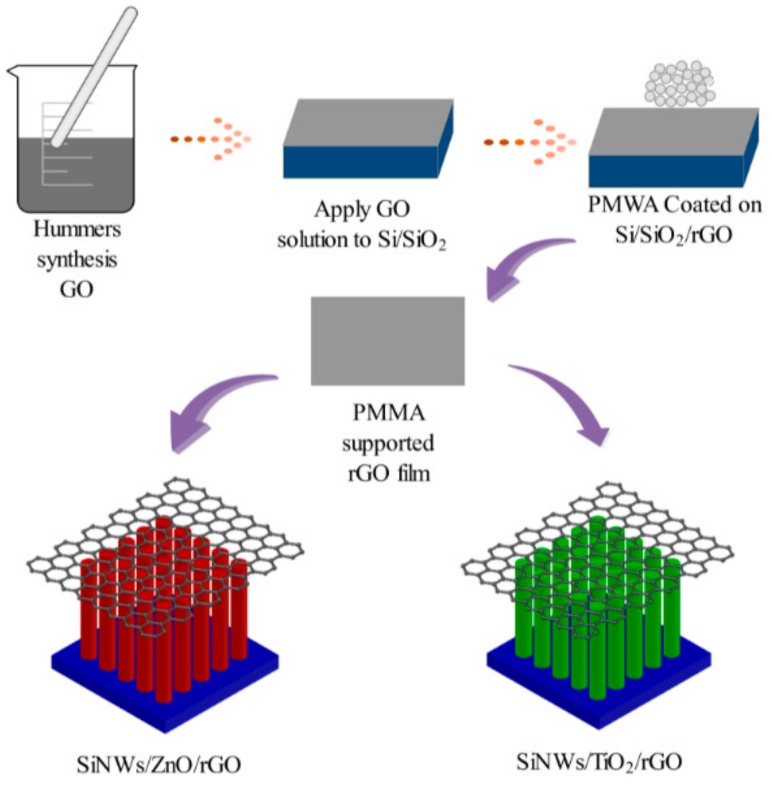
Schematic of the device fabrication process.

The design process of the Schottky structure sensor is shown in Figure 1. Firstly, the SiNW array was constructed in a stainless steel autoclave filled with an etched nitrate solution; the obtained sample was washed with deionized water, dried, and then subjected to oxygen plasma treatment to obtain sufficient hydroxyl groups [19,20]. Next, we dissolved zinc acetate dehydrate (Biotech Bioengineering Co., Ltd, Shanghai, China) in methanol, gradually added it to KOH, and then immersed the SiNW array in the solution to obtain a SiNW array decorated with ZnO nanoparticles. Then, we took TiCl_4_ which had been stored in the refrigerator, quickly injected it into a sealed bottle filled with ice, sealed the bottom with deionized water, and quickly shook it until the ice melted. We immersed the SiNW array into the TiCl_4_ solution to obtain a TiO_2_-modified SiNW array [21,22]. Finally, Hummers-synthesized GO was overcoated onto the SiNW array to construct the electrode with reduced graphene oxide (rGO) on the top surface [23,24]. 

### 2.2. Characterization

**Figure 2:**
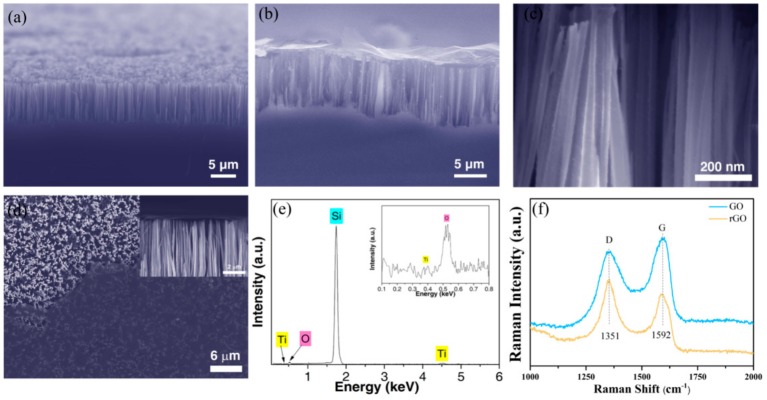
(**a**) Cross-section FESEM image of the SiNW array; (**b**) FESEM cross-section of the SiNW/ZnO/rGO heterojunction; (**c**) SEM image of SiNWs decorated with TiO_2_ nanoparticles; (**d**) SEM image of the SiNW/TiO_2_/rGO Schottky heterojunction; the inset is a side screenshot; (**e**) XPS of the SiNW interface layer decorated with TiO_2_ nanoparticles; (**f**) Raman spectra of GO and rGO films.

Scanning electron microscopy (FESEM, ZEISS SUPRA 55VP, Carl Zeiss Group, Oberkochen, Germany) was used to check the surface morphology of the prepared sensor. The surface properties were studied by X-ray photoelectron spectroscopy (XPS, ESCALAB250, Thermo VG Scientific, West Sussex, UK). The structural characteristics of the sensor were characterized by Raman scanning spectroscopy (Horiba Jobin Yvon, LabRAM HR800, S.A.S., Longjumeau, Cedex, France). Figure 2a is a cross-section FESEM image of the SiNW array. It can be seen that a highly ordered, vertically aligned silicon nanowire array with a length of about 10 μm was obtained. Figure 2b is a FESEM cross-sectional view of the SiNW/ZnO/rGO heterojunction. It can be seen from the figure that the top of the silicon nanowires decorated with ZnO nanoparticles was closely attached to rGO. This tightly attached structure will significantly facilitate the transfer and transportation of electrons in water molecules. Figure 2c is an enlarged SEM image of SiNWs decorated with TiO_2_ nanoparticles. It can be seen from the figure that the surface of the SiNWs was successfully decorated with TiO_2_ nanoparticles. Figure 2d is a SEM image of the SiNW/TiO_2/_rGO Schottky heterojunction, and the inset is a side screenshot. The successful formation of the Schottky heterostructure involves a large number of defects on the surface, resulting in a significant increase in activity and improving the amount of water absorption. Because the doped TiO_2_ is relatively small, the existence of TiO_2_ cannot be directly seen in Figure 2c, so XPS characterization was performed to demonstrate that the SiNW was decorated with TiO_2_ nanoparticles. In Figure 2e and the inset figure, the appearance of characteristic peaks of titanium and oxygen confirms the successful surface assembly of TiO_2_. Figure 2f shows that GO and rGO have obvious peaks at 1351 cm^−1^ and 1592 cm^−1^, corresponding to the D and G peaks, respectively. After reduction of graphene, both the D and G peaks shifted downward. I(D)/I(G) is the intensity ratio of the D-peak and G-peak, the D-peak represents defects in the C atom lattice, and the G-peak represents the in-plane expansion and contraction of C atom sp2 hybrid vibration. The I(D)/I(G) value of rGO (1.12) is clearly greater than that of GO (0.91), revealing the formation of new sp^2^.

### 2.3. Humidity Testing

**Figure 3:**
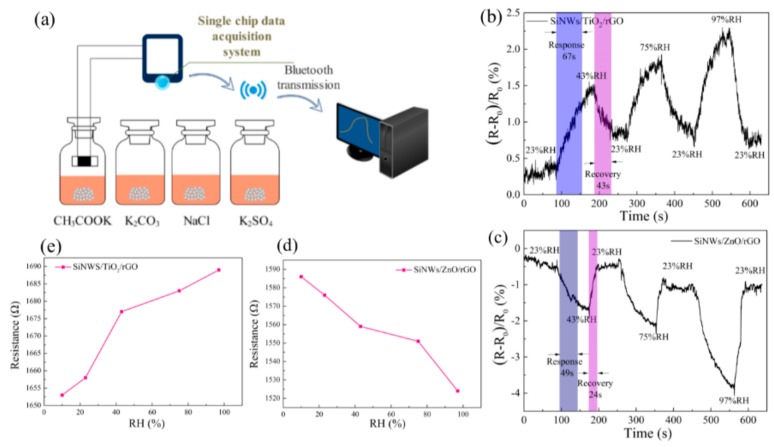
(**a**) Schematic diagram of the humidity sensing experimental setup; (**b**)–(**c**) the response curves of the two types of sensors to four levels of humidity; (**d**)–(**e**) the resistance of the two sensors as a function of humidity.

We used a classic saturated salt solution method to test the sensitivity of the developed sensors at different humidity levels [25,26]. First, we configured the saturated salt solution of CH_3_COOK, K_2_CO_3_, NaCl, and K_2_SO_4_ (Tianjin Huihang Chemical Technology Co., Ltd, Tianjin, China) at room temperature to simulate environmental relative humidity levels of 23%, 43%, 75%, and 97%, as shown in Figure 3. Then, we purified the gas cell with dry air (DG2000 and AT160/60CS, Soret, Beijing, China) to obtain the characteristics of the sensor at 10% relative humidity. All experiments were performed at room temperature to reduce random errors. The change in resistance of the sensor in different relative humidity environments was measured to study the humidity sensitivity characteristics and the corresponding response time.

### 2.4. Electrical Characterization

The sensor was secured to the Printed Circuit Board (PCB) with conductive silver slurry, and copper wire electrodes were placed at both ends of the circuit board. The experimental data acquisition was performed using a miniature hardware terminal based on an STM32F030 microcontroller (ST Microelectronics, Shanghai, China), which was controlled by an Android phone connected via Bluetooth. The resistance values were obtained by the voltage-divider circuit, and were collected and plotted on the phone after filtering and smoothing (see details in Video S1, Supporting Information). We define the sensitivity S of the original thin film according to the rate of change of resistance:

(1) $$S=\frac{R-R_{0}}{R_{0}}$$

where R is the resistance value of the sensor in a humid environment, and R_0_ is the initial value of the sensor under dry conditions. The response time and recovery time of the sensor were defined as the time it takes for the resistance of the sensor to change from the initial value to 90% of the stable offset.

## 3. Results and Discussion

### 3.1. Humidity Sensing Performance

The two Schottky sensors were employed to perform humidity sensing measurements at room temperature, and the results are displayed in Figure 3. It is important to consider the response and recovery times of the sensors to a given environmental humidity. Figure 3b shows that the response time of SiNW/TiO_2_/rGO was 67 s and the recovery time was 43 s in the 10-minute humidity sensing test, and in Figure 3c, the SiNW/ZnO/rGO had a response time of 49 s and a recovery time of 24 s. The obtained values are in the same order of magnitude as those of previous reported humidity sensors [27]. While conducting experiments from high humidity to low humidity, the recovery speed of the two sensors was relatively slow, which is a problem to be solved. Figure 3d,e shows the respective resistance values of five groups under 10%, 23%, 43%, 75%, and 97% humidity conditions, and the measured resistance value at 10% humidity was taken as R_0_. It can be seen that the resistances of two sensors have opposite trends as the humidity changes: The resistance of the SiNW/TiO_2_/rGO sensor increases with increasing humidity, while that of the SiNW/ZnO/rGO sensor decreases with increasing humidity. 

### 3.2. Humidity Sensing Mechanism

TiO_2_ and ZnO can regulate the performance of a Schottky-structure-based sensor, while water molecules have a p-type doping effect on graphene and can regulate the Fermi level of graphene. The water molecules act as electron donors; when the humidity increases, additional electrons enter the ZnO conduction band, leading to an increase in the carrier concentration and the conductivity of the nanostructure [28,29]. The value of Δ*R*/*R*_0_ increases with increasing humidity, well consistent with a previous report [30]. TiO_2_ increases the oxygen vacancies on the sensor surface and promotes the separation of water molecules, making the resistance value increase with increasing humidity [31]. At the same time, according to Hard Soft Acid Base (HSAB) theoretical analysis, hard acid easily interacts with hard base, and soft acid easily interacts with soft base. In addition, it is known that water molecules are hard bases, and Ti^4+^ and Zn^2+^ are acids. The electrons are provided by water molecules, and acids accept electron pairs, so as the humidity increases, charge can be transferred inside the sensor [32]. Under high-humidity conditions, some water molecules often remain on the surface of the moisture-sensitive material by chemical or physical adsorption, which makes the two materials desorb water at a slow rate during the experiments from high humidity to low humidity. 

### 3.3. Human Breathing Test Results

**Figure 4:**
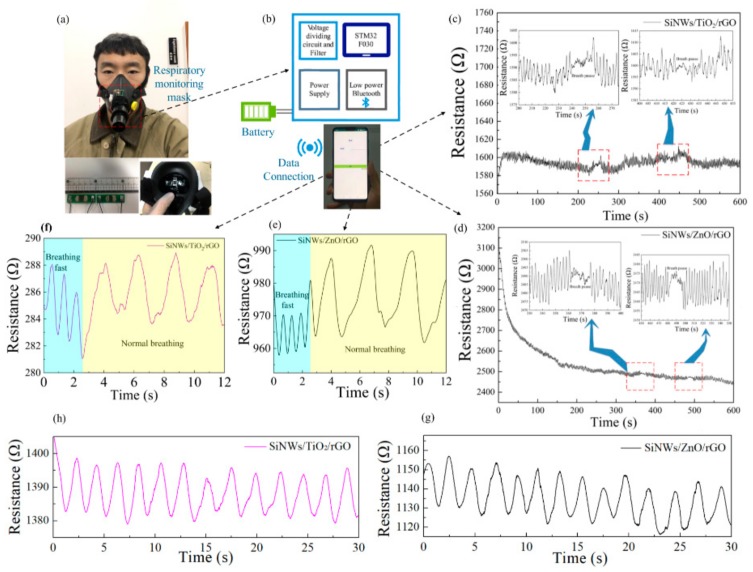
(**a**,**b**) Respiratory monitoring mask system. (**c**,**d**) 10-minute breath monitoring with the two types of sensors. (**e**,**f**) The resistance variation response to normal and fast breathing. (**g**,**h**) Response of both sensors measured after 30 days.

In order to evaluate the humidity sensing properties of the two SiNW-based sensors in practice, a breathing monitoring experiment was performed on volunteers wearing a sensor-equipped mask for up to 10 min, both breathing normally and simulating the situation of apnea (holding breath). As shown in Figure 4c,d, the waveforms have distinct differences during breath holding in the resistance change slack when compared to normal breathing conditions, as displayed within the time periods of 360–375 s, 480–495 s and 240–255 s, 420–435 s. This facilitates the clinical monitoring of patients. At the beginning of the tests, the SiNW/ZnO/rGO had a significant resistance baseline drift; we will solve this problem in the future. Figure 4e,f shows simulated fast breathing and normal breathing, respectively. The changes in waveform profiles and periods of humidity response are significantly different, which also confirms the feasibility of monitoring human breathing patterns. After 30 days, the responses of the two sensors were measured again, and the results are shown in Figure 4g,h; the sensitivity remained very good, indicating the reusability of the sensor.

## 4. Conclusions

In summary, we presented two Schottky structure sensors and demonstrated their good sensitivity and good repeatability in human breathing monitoring. The response times of SiNW/ZnO/rGO and SiNW/TiO_2_/rGO were 49 s and 67 s, and the recovery times were 24 s and 43 s, respectively; the response and recovery times of SiNW/ZnO/rGO were better than those of SiNW/TiO_2_/rGO. For respiratory monitoring, SiNW/ZnO/rGO sensors are more suitable for human respiratory monitoring because of their faster response and better sensitivity. In short, these sensors based on Schottky structures have great potential for the development of future portable respiratory monitoring systems, and they also have certain implications for the development of new moisture-sensitive materials.

## Supplementary Materials

The following are available online at https://www.mdpi.com/1996-1944/13/2/430/s1, Video S1: Android phone data acquisition and processing operation demo.
